# Reciprocal Supportive Interplay between Glioblastoma and Tumor-Associated Macrophages

**DOI:** 10.3390/cancers6020723

**Published:** 2014-03-26

**Authors:** Wenchao Zhou, Shideng Bao

**Affiliations:** Department of Stem Cell Biology and Regenerative Medicine, Lerner Research Institute, Cleveland Clinic, Cleveland, OH 44195, USA; E-Mail: zhouw@ccf.org

**Keywords:** glioblastoma multiforme (GBM), tumor-associated macrophage (TAM), infiltration, microglia, immune therapy

## Abstract

Glioblastoma multiforme (GBM) is the most lethal and aggressive type of primary brain malignancy. Failures of the traditional therapies in treating GBMs raise the urgent requirement to develop new approaches with more responsive targets. The phenomenon of the high infiltration of tumor-associated macrophages (TAMs) into GBMs has been observed for a long time. Regardless of the limited knowledge about TAMs, the high percentage of supportive TAM in GBM tumor mass makes it possible to be a good target for GBM treatment. In this review, we discussed the unique features of TAMs in GBMs, including their origin, the tumor-supportive properties, the secreted cytokines, and the relevant mechanisms. In addition, we tried to interpret the current understandings about the interplay between GBM cancer cells and TAMs. Finally, the translational studies of targeting TAMs were also described.

## 1. Introduction

Decades have passed since cancer has become a major subject for life science studies. However, it was not until recently that people realized the cellular heterogeneity and hierarchy of tumors. Within a tumor, many non-neoplastic cells, such as stromal cells, endothelial cells, and immune cells, assist tumor growth by producing various growth factors and pro-angiogenic cytokines [1,2,3]. Glioblastoma multiforme (GBM) is the most common and aggressive type of primary brain tumor [4]. As the World Health Organization grade IV astrocytic tumor, GBM affects 3 per 100,000 individuals annually [5]. Despite aggressive treatment, the median survival of these patients is less than 16 months [6]. Many factors contribute to the malignant progression of GBM and its resistance to current therapeutics. One of the most commonly detected phenomena in GBM is the abundant macrophage infiltration without apparent phagocytic activity [7]. Accumulating evidences have suggested that the tumor-associated macrophage (TAM) infiltration can be linked to the poor prognosis in GBM [8]. However, there are controversial reports about the origin, the recruitment, and the functions of the tumor-associated macrophages in GBM. Furthermore, the interplay between tumor-associated macrophages and the host GBM cancer cells is poorly understood. In this review, we try to summarize the current understanding of the above mentioned aspects of tumor-associated macrophages in GBMs. Some clinical trials along with preclinical studies concerning TAMs were also discussed.

## 2. Infiltration of TAMs in Glioblastoma

Lymphocytic infiltration in glioblastomas has been noticed since the early 1970s [9]. When the infiltrated mononuclear cells in human glioblastomas were determined by immunostaining of cell surface differentiation antigens, macrophages were identified along with T cells and B cells. The number of other lymphoid cells showed no correlation to tumor grades, but there was a significant increase in the number of macrophages in high-grade astrocytomas, *i.e.*, GBMs [10,11]. Quinolinic acid, which is only produced by macrophages and microglia within the brain [12], was stained in immune cells infiltrated in GBMs. Variable numbers of quinolinate immunoreactive cells were observed in and around the tumors, but the majority of the quinolinate positive cells presented within tumors [13]. Thereby unlike other non-neoplastic cells, e.g., astrocytes that locate mostly external to the tumors, the TAMs infiltrated and located within tumors with no differences between central and peripheral tumor areas [14]. Other studies using macrophage lineage immunohistochemical markers, including HLA-DR and Iba1, proved the infiltration of macrophages within tumor areas and suggested a strong correlation of macrophages with tumor malignancy [14,15]. Interestingly, the infiltrated TAMs were morphologically variable, including round, complex, rod-shaped, and sparsely dendritic cells [13]. Noticeably, macrophage infiltration may be GBM subtype dependent. Correlation between the enrichment of macrophage-related genes and the poorer survival occurred in adult but not in pediatric GBMs [16]. Likewise, significantly higher numbers of macrophages were detected in adult mesenchymal GBMs compared to non-mesenchymal tumors [16]. The macrophage infiltration was not only detected in human primary GBMs, but also in dog GBM and human xenograft GBM models in immune-deficient mice, and syngeneic GBMs in immune-competent rodents [17], suggesting a universal role of tumor-associated macrophage in GBM tumorigenesis.

## 3. Origins of the TAMs in GBM

Compelling evidence in several different solid tumors indicated that tumor-associated macrophages come from monocyte recruitment [18,19,20]. However, the situation in brain tumors is more complex. This is largely due to the unique immune cells, *i.e.*, microglia, which exclusively reside in the brain. Microglia is an abundant cell population in the CNS, comprising 5% to 20% of the total glial cell population [21,22]. It has been proposed that circulating microglia precursors, derived from mesodermal hematopoietic cells, enter the developing brain during perinatal stages and transform into microglia cells [23]. Mature microglia reside in adult brains and share a variety of cell surface markers with monocyte-derived macrophages, including CD11b [24] and Iba1 [15]. Therefore it’s hard to distinguish microglia from macrophages in GBM tumors. Considering the huge number of microglia in the proximity of GBM tumors, it’s plausible that the resident microglia serve as a major resource of tumor-associated macrophages. In fact, many studies used “microglia/macrophages” to denote TAMs in GBMs. Recently, the generation of CX3CR1(+/GFP)CCR2(+/RFP) knock-in fluorescent protein reporter mice made it possible to investigate monocyte subset trafficking. The results suggested that CX3CR1 and CCR2 distinguished infiltrating macrophages from activated microglia [25,26], indicating that microglia and macrophages are different populations with distinct specific surface antigens. Although the fluorescent protein reporter mouse has not been used in GBM study so far, it provides a tool to track down the origin of TAMs in GBMs. Meanwhile, some evidence suggests different functions of microglia and macrophages in GBM tumor microenvironment. Immunohistochemical staining of human GBM biopsies with a series of different monocyte/macrophage markers demonstrated the distinct distribution of macrophages and activated microglia within brain tumors: macrophages in the tumor center, microglia at the border zone or adjacent brain tissue. [14]. Furthermore, in CX3CR1−/− mice with intracranial gliomas, the number of infiltrated TAMs was similar to that in CX3CR1+/− mice, along with a slight increase in tumor growth in CX3CR1−/− mice [27], suggesting that microglia has little contribution in TAM infiltration and the GBM tumor growth. Finally, in human GBM patients, the common CX3CR1-I249 allele has been shown to be an independent favorable prognostic factor in survival analysis, indicating that CX3CR1+ microglia may have a role in tumor progression [28]. Taken together, microglia is different from monocyte-derived macrophages and whether it attenuates or promotes GBM tumor growth is still questionable. 

Given the limited evidence showing infiltration of microglia in GBMs, monocytes are more likely to be the precursor cells of the infiltrated TAMs. The monocyte-derived macrophages have been reported in several solid tumors [29,30]. However, it’s difficult to determine the fraction of monocyte-derived macrophages in TAMs. Interestingly, glioblastoma-associated peripheral blood monocytes themselves may be distinct from those of healthy individuals. For example, a much higher amount of epidermal growth factor was detected in peripheral blood monocytes isolated from patients with malignant gliomas when compared to those from healthy controls [31]. In addition, circulating CD11b+ cells sorted from patients with GBM were found to markedly suppress normal donor T-cell function in coculture [32]. Therefore, although the contribution of monocytes to TAM infiltration in GBMs remains to be clarified, monocytes in GBM patients may be endowed with tumor supportive features in the early stage of differentiation before and after infiltration into GBM tumors. 

Finally, it’s worth mentioning the possibility that part of TAMs may come from cells other than microglia or monocytes. It has been demonstrated that human malignant astrocytes express macrophage phenotype according to the staining of the macrophage markers CD11b, CD68 and HAM56 [33]. Thus, in GBMs, the term “tumor-associated macrophages” could be used to denote no specific immune cell subpopulations but rather a collective of macrophage-marker expressing cells infiltrated into tumor areas, regardless of their origins.

## 4. Properties of TAMs in Glioblastoma

Traditionally, macrophages function as phagocytes in innate immunity, or as antigen presenting cells in adaptive immunity. Based on such knowledge, it was hypothesized that tumor-associated macrophages may function to eliminate cancer cells and in turn suppress tumor progression. The presumption was supported by some experimental facts. Immunocytochemistry demonstrated the prominent expression of Adenosine A1 receptor (A1AR) in TAMs compared to the microglia in the unaffected brain tissue. In organotypical brain slice cultures, A1AR agonists suppressed tumor growth. Intracranially implanted glioblastoma grew more vigorously in A1AR-deficient mice. Thus, A1AR positive TAMs exert an adenosine dependent, tumor suppressive function in GBM growth [34]. Besides, the immune-modulatory nonclassical molecules HLA-G and HLA-E were found in TAMs in a majority of glioblastomas. It was speculated that the expression of these molecules by activated macrophages may play a role in the anti-tumor immunity in the development of glioblastoma [35].

On the other hand, the majority of TAMs in GBMs showed no phagocytosis. Double staining of the microglia/macrophages by quinolinate and the phagocytes by lectin histochemistry demonstrated only partial co-distribution of the two markers [13]. Similarly, immunostaining showed that in contrast to leukocytes outside the tumor, which were activated and expressing class II major histocompatibility antigens, TAMs within the tumor parenchyma or at the tumor’s edge were negative for these antigens, indicating no antigen presenting activity in TAMs [36]. Surprisingly, in GBMs treated with oncolytic viruses, both phagocytic marker CD68 and tumor suppressive macrophage marker CD163 were elevated in subpopulations of TAMs. While the TAMs function to antagonize the anti-tumor effect of oncolytic viruses in GBMs, the CD68+ population may clear the apoptotic cancer cells in response to oncolytic treatment, whereas the CD163+ population may promote the growth of the remaining uninfected cancer cells [37,38]. In general, TAMs in GBMs are not likely to be classically activated macrophages that are supposed to attenuate tumor growth.

Macrophages can be phenotypically polarized by the microenvironment to become M1 or M2 subtypes [39]. Originally defined by secretion of IL-2 or IL-10, which in turn activates Th1 or Th2 cells, now the concepts of M1/M2 subtypes basically represent tumor suppressive or tumor supportive macrophages, albeit a little bit oversimplified [40]. Several cell surface markers have been suggested to distinguish the M1/M2 macrophages, including CD163, Fizz1, Arg1 and MHCII [41,42,43,44,45]. When stained by these markers, TAMs in glioblastomas manifested strong M2 macrophage characteristics. For instance, the M1 marker MHCII was common in lower grade astrocytomas. Meanwhile, although a large portion of immune cells expressing macrophage markers still present in glioblastomas, the MHCII immunoreactivity was dramatically reduced [46]. In contrast, the expression of the M2 macrophage marker iNOS was detected in infiltrating macrophages in human glioblastomas [47]. Likewise, the number and the ratio of macrophages with positive staining for CD163 and CD204, two well-known markers for M2 macrophages, were correlated with the histological grade of the gliomas [41]. According to these observations, a large proportion of the TAMs in GBM should be of M2 tumor supportive subtype.

## 5. TAMs Support GBM Tumor Progression

Besides the positive staining of M2 subtype surface markers, several lines of evidence underscore the supportive role of TAMs in GBM tumor progression. TAMs can promote proliferation of GBM cancer cells by secretion of relevant cytokines. For instance, *in situ* hybridization revealed that interleukin 10 (IL-10) mRNA production is restricted to TAMs *in vivo*. IL-10 increases glioma cell proliferation *in vitro* and its expression has been found to be correlated with the extent of malignancy in gliomas [48]. Similarly, two members of the VEGF family, VEGF-C and VEGF-D, were found to be co-stained with CD163+ TAMs in GBMs [49]. IL-6 expression was localized in macrophages too [50]. In addition, the co-chaperone stress inducible protein 1 (STI1), which increased the proliferation of GBM cells *in vitro*, was also reported to be synthesized and secreted by TAMs [51].

TAMs could also facilitate GBM tumor growth by promoting neo-vascularization. The positive correlation between macrophage infiltration and the vascular density in human gliomas has been observed in several studies [52,53,54]. Indeed, heme oxygenase-1 (HO-1), a rate-limiting enzyme in heme catabolism that associated with neoangiogenesis, was predominantly observed in TAMs. Consistently, a correlation between HO-1 mRNA level and macrophage infiltration and vascular density has been found in human gliomas [55,56]. Another angiogenic enzyme, thymidine phosphorylase (TP), was also predominantly detected in TAMs. Given that high TP expression was observed in most of malignant GBMs and there’s a good correlation between TP-expression and the microvessel density, it’s highly possible that tumor-associated macrophages benefit vascularization via TP in GBMs [52,53]. Moreover, the expression of Platelet-derived endothelial cell growth factor (PD-ECGF) in GBMs was colocalized with the macrophage marker HAM-56. In the meantime, a positive correlation between PD-ECGF and the degree of stromal vascularity was detected in GBM [57]. Although TAMs seem to promote angiogenesis in GBMs, people should be cautious to interpret the correlation between vessel density and the TAM infiltration in GBMs. Since a large proportion of TAMs are derived from peripheral blood monocytes, a high vessel density could simultaneously facilitate and result in a high TAM infiltration. From such aspect, the vessel density determines the TAM infiltration.

In addition, TAMs are involved in invasiveness of GBMs. Microglia strongly enhanced glioma invasiveness in a co-culture system. Such invasion-promoting activity was lost in glioma cells lacking TGF-beta receptor, indicating the implication of TAM-secreted TGF-beta in GBM cancer cell invasion [58]. The TAM-secreted STI1 was also reported to be involved in TAM-induced GBM invasion [59]. Interestingly, microglia migration itself may stimulate the invasion of glioblastoma cells. In an *in vitro* co-culture system, inhibition of microglia migration by blockage of colony stimulating factor 1 receptor (CSF-1R) strongly suppressed glioblastoma invasion [59]. 

Whereas TAMs do not show many traditional immunocyte properties, they can interfere with the functions of other immune cells to help GBM tumor progression. TAMs produce interleukin-1 (IL-1), an immunoregulatory polypeptide, to decrease the expression of cell surface antigen HLA class II in glioblastoma cancer cells, partially through impairing the cell response to IFN-gamma [60]. Thereby, macrophages help GBM cancer cells to escape from being targeted by T cells. Furthermore, evidence suggests that TAMs release some soluble factors to promote apoptosis of activated T-cell, although the effective components of these soluble factors were not elucidated [61].

Finally, TAMs contribute to resistance to radio-chemotherapy of GBMs. In irradiated relapses, there’re significantly more TAMs expressing MRP-14, an immunosuppressive marker, compared to untreated GBM relapses, suggesting the increase of de-activated or immature macrophages after radio-chemotherapy. In contrast, a significant increase of CD68 expressing phagocytic macrophages was observed in patients without postsurgical treatment, but not in those with radio-chemotherapy [62]. It’s possible that compared to the regular activated macrophages, the immunosuppressive TAMs are more resistant to radio-chemotherapy and in turn assist GBM relapse. Taken together, TAMs support GBM tumor progression and relapse by promoting proliferation and invasion of cancer cells, elevating neo-vascularization, and suppressing anti-immunity in GBMs. 

## 6. GBM Cancer Cells Recruit Tumor-Associated Macrophages

The infiltration of TAMs in GBM can be, at least in part, ascribed to GBM cancer cells. Many cytokines secreted by GBM cancer cells may function to recruit TAMs. For example, siRNA mediated reduction of glial cell-derived neurotrophic factor (GDNF) in mouse glioma cells diminished attraction of TAMs *in vivo* [63]. Another cytokine, granulocyte-macrophage colony-stimulating factor (GM-CSF), is also involved in TAM recruitment. The number of infiltrating Iba1+ TAMs was reduced in murine gliomas depleted of GM-CSF. Consequently, knockdown of GM-CSF in GL261 glioma cells strongly reduced growth of intracranial gliomas and extended animal survival [64]. Vascular endothelial growth factor (VEGF) is a well-known cytokine in GBM that promotes angiogenesis [65,66]. However, VEGF may also promote TAM recruitment, since an antibody to FLT-I, the VEGF receptor, diminished the accumulation of tumor infiltrating macrophages [67]. Besides, other cytokines from GBM cancer cells, including macrophage inhibitory cytokine-1 (MIC-1), transforming growth factor (TGF-beta1), and soluble colony-stimulating factor (sCSF), also demonstrated the capacity to attract monocytes/macrophages [68]. Therefore, multiple soluble factors produced by GBM cancer cells contribute to TAM recruitment.

Recruitment of TAMs by GBM cancer cells can be enhanced under certain pathophysiological conditions. In the hypoxic regions within GBM tumors, there’s an increased expression of stromal-derived factor-1 (SDF1), which is able to recruit bone marrow-derived CD45+ myeloid cells containing Tie2+, VEGFR1+, CD11b+, and F4/80+ subpopulations [69]. These CD45+ cells may have the capacity to initiate angiogenesis by increasing VEGF bioavailability through its matrix metallopeptidase-9 (MMP-9) activity [69]. The upregulation of SDF1 is partially regulated by HIF1alpha, the direct effector of hypoxia. In the absence of HIF1alpha, SDF1 levels decrease, and fewer BM-derived cells are recruited to the tumors [69]. HIF1alpha was induced in the irradiated tumors. Similar to hypoxia, irradiation induced recruitment of bone marrow-derived cells (BMDCs) into the tumors and this BMDC influx contributed to restore the GBM tumor growth [70]. Blocking this influx by pharmacologic inhibition of HIF1alpha or of the SDF-1/CXCR4 interaction prevented tumor recurrence [70]. The HIF1alpha/SDF-1 induced TAM recruitment in GBMs is of extreme importance when predicting the outcome of anti-tumor therapeutics. Recently, inhibition of neovascularization by VEGF/VEGFR neutralization has become a popular approach in treating GBMs. However, multi-targeted VEGFR inhibition rapidly created a vascular gradient and induced tumor hypoxia, accompanied by re-infiltration of TAMs. The increase of the CD11b+ TAM infiltration was associated with tumor progression as well as the aggressive mesenchymal features and the increased expression of stem cell marker [71]. Clinically, the increased number of CD11b+ cells correlated with poor overall survival among patients who first received anti-angiogenic therapy at recurrence [72]. All these facts suggest that GBM tumors can resist radio-chemotherapy by recruitment of tumor-associated macrophages.

The cytokines in GBMs usually have multifaceted functions, so the real effects of these cytokines *in vivo* are far from clarified. The monocyte chemoattractant protein-1 (MCP-1) provides a good example. Constitutive expression of MCP-1 was detected in glioblastomas and astrocytomas [73]. It was reported that MCP-1 plays a role in TAM infiltration because anti-MCP-1 antibodies neutralized monocyte chemoattraction to tumor cyst fluids from glioblastomas *in vitro* [74]. However, one of the MCP-1 receptors, CC chemokine receptor-2A (CCR2A), is frequently overexpressed in glioblastoma cancer cells and inhibition of MCP-1 synthesis suppressed migration of CCR2A-overexpressing glioma cells. Therefore, MCP-1 may exert its tumor supportive activity via directly affecting cancer cell migration [75]. Besides CCR2A, a systematic analysis of cytokine receptors revealed that the majority of glioblastomas showed the expressions of IL-1 receptors (IL-1RI and IL-1RII), tumor necrosis factor receptors (p75TNFR and p55TNFR), interferon alpha/beta/gamma receptors, IL-8 receptor, M-CSF receptor and stem cell factor receptor [76]. Thus, it’s possible that rather than recruiting TAMs, most of the cytokines produced by GBM cancer cells function to trigger autocrine or paracrine signaling pathways in cancer cells, although they may exhibit chemoattactive activities to monocytes/macrophages *in vitro*.

Interestingly, some soluble factors in GBMs may suppress the TAMs attraction. The soluble decoy receptor 3 (DcR3) is highly expressed in GBM cancer cells. However, the tumors derived from cancer cells with ectopic DcR3 expression showed a substantial decrease in infiltrated immune cells, including macrophages and T cells [77]. This phenomenon suggested that DcR3 produced by GBMs may confer suppression of macrophage infiltration. On the other hand, in intratumoral lipid environments, free docosahexaenoic acid was found to induce macrophage apoptosis [78]. It will be interesting to explore whether these factors function as pan-inhibitory regulatory mechanisms for all TAM infiltration, or they distinctly suppress some categories of TAMs to achieve a balanced TAM subtype ratio in the GBM tumor environment.

## 7. GBM Cancer Cells Endow TAMs with Tumor Supportive Characteristics

One of the critical questions concerning TAMs in GBMs is the gain of tumor supportive characteristics by TAMs. Although GBM is composed of many different cell types, so far most studies have been focused on the influences from host tumor cells on TAMs. It was reported that fluid from GBM tumors induced morphological transformation of microglia and activate MAPK signaling *in vitro* with absence of pro-inflammatory factors, suggesting that GBM cancer cells secreted some soluble factors to alternate macrophage characteristics [79,80]. In fact, the GBM-conditioned medium inhibited macrophage phagocytosis, induced the secretion of the immunosuppressive cytokines interleukin-10 (IL-10) and TGF-beta1 by the macrophages, and enhanced the capacity of macrophages to inhibit T-cell proliferation, thereby polarizing the macrophages to an M2 phenotype [68]. The induction of M2 phenotype may occur in the earlier stage. When healthy donor human CD14+ monocytes were cultured with human glioblastoma cell lines, the glioblastoma-conditioned monocytes had immunosuppressive features, including reduced CD14 (but not CD11b) expression, increased secretion of interleukin-10, TGF-beta, and B7-H1 expression, decreased phagocytic ability, and increased ability to induce apoptosis in activated lymphocytes. Control monocytes co-cultured with normal human astrocytes didn’t show these features [81]. These facts indicated that GBM cancer cells have the capacity to transform monocytes/macrophages into M2 immunosuppressive subtype. 

Several studies have demonstrated the education of macrophages by GBM conditioned medium and soluble factors secreted by GBM cancer cells were supposed to be responsible. However, so far few cytokines produced by GBMs can be linked to the gain of M2 subtype by TAMs. Macrophage colony-stimulating factor (M-CSF), which strongly induces M2 polarization of macrophages, was significantly correlated with histological malignancy and with the proportion of M2 microglia/macrophages *in vivo* [41]. Surprisingly, M-CSF is not a common cytokine secreted by GBM cell lines *in vitro*. The conflict can be explained by the hypothesis that secretion of the M2-inducing factors by GBM may have to be triggered by outside stimulations. It was reported that when stimulated by TNF, enhanced production of IL-6, IL-8, GM-CSF, prostaglandin E2 (PGE2) and manganous superoxide dismutase (Mn-SOD) were observed in GBM cells [82]. Besides, GBM-mediated inhibition of macrophage phagocytosis was potentiated by hypoxia [83]. In addition, cytomegalovirus (CMV), which has been ubiquitously detected within high-grade gliomas, stimulated GBM-mediated induction of human monocytes to assume an M2 immunosuppressive phenotype as manifested by down-modulation of the major histocompatibility complex and costimulatory molecules [84]. Of note, it was also suggested that direct contact between monocytes and glioblastoma cells is necessary for complete induction of the immunosuppressive features [81].

On the other hand, the ability of GBM to induce M2 subtype macrophages may partially be ascribed to its lack of signals necessary for macrophage activation. For instance, granulocyte-colony-stimulating factor (G-CSF), which is capable of inducing mature macrophage, is highly expressed in astrocytoma WHO grades I and II and reactive brain tissue, but there’s low expression of G-CSF in astrocytoma WHO grade III, and none in glioblastoma [85]. 

Although the cytokines responsible for M2 TAM induction are largely unknown, there’re some clues for the upstream regulators. The inhibition of phagocytosis and the secretion of IL-10 were reversed when the signal transducer and activator of transcription 3 (STAT3) pathway was blocked in the GBM cells [68]. Furthermore, hypoxia-potentiated immunosuppression by M2 TAMs was down-regulated by inhibition of STAT3 and its downstream effectors HIF1alpha [83]. Interestingly, it was reported that GBM tumor cells up-regulated STAT3 and STAT5 signaling pathways in monocytes/macrophages, but did not interfere with the TLR- or CD40-induced activation, to suppress monocyte activation and achieve immunosuppression [86]. Therefore, it’s possible that some signaling pathways regulate the cytokine secretion both in GBM cancer cells and TAMs to constitute an immunosuppressive environment in GBMs.

## 8. TAM-Related Immunotherapy in GBM Treatment

Treating cancer by enhancing immune responses against tumor cells is an attractive idea and it has been applied in GBM treatment for a long while. Several strategies targeting different immune cells were proposed and tried in GBM patients. Basically, immune cells (lymph nodes, dendritic cells, *etc.*) from patients were stimulated with GBM tumor antigens (intact cells, irradiated cells, cell surface antigen, or cell lysate) to endow them with some kinds of immune memory [87,88,89,90,91]. These *ex vivo* activated immune cells were then injected back to the patients to elicit anti-tumor immune responses. Some modifications were performed in these therapies, including combination of the antigen with cytokines to enhance the immune cell activities [87], or using a subpopulation of GBM cancer cells, like cancer stem cells (CSCs), as the antigen [92]. However, the outcome of these immunotherapies was not optimistic. Although some patients demonstrated partial regression of residual tumor [87], most recipients didn’t benefit from these treatments [93,94]. The unsatisfactory results may be ascribed to the unique feature of GBMs. In contrast to other solid tumors, failure to present tumor antigens is not a likely impediment to immunotherapeutic strategies against malignant gliomas since GBMs frequently express both human leukocyte antigen Class I and II molecules. Instead, few B7 co-stimulatory molecules, which are required for CD4+ T-cells activation, are detected in GBMs [95]. Thus, immunotherapeutic strategies need to overcome low levels of B7 co-stimulation. On the other hand, in many cases, immunotherapies did elicit an enhanced TAM infiltration, but no anti-tumor immunity was detected [93,94,96]. The immunosuppressive phenotype of the infiltrated macrophages in GBMs, along with the capacity of GBM cancer cells to induce such compromised immunity, may count for the failure of the immunotherapies in GBMs.

Preliminary studies in GBM rodent models have proved the potential to treat GBMs by depleting TAMs. Folate receptor beta (FR-beta) was expressed on macrophages in human glioblastomas and rat C6 gliomas. Targeting tumor-associated macrophages in C6 glioma xenografts in nude mice with a recombinant immunotoxin to FR-beta significantly depleted TAMs and reduced tumor growth [97]. In addition, propentofylline (PPF), an atypical methylxanthine, significantly decreased tumor growth in a CNS-1 rat model of GBM by targeting TAMs but not tumor cells [98,99]. Noticeably, it has been reported that systemic depletion of Tregs cells resulted in improved long-term survival if treatment started 15 days after tumor implantation. Such improvement was not observed if depletion protocol began 24 days after tumor implantation [100]. It’s highly possible that the same time-window effect, probably from tumor burden, may hamper the treatment of GBM by depletion of TAMs.

The percentage of TAMs infiltrating glioblastoma can reach up to 30% of tumor mass [101]. Therefore it could be extremely difficult to eliminate the TAMs. A more attractive hypothesis is to restore the anti-tumor activities in TAMs to destroy the GBM cancer cells. Several preclinical studies have taken this approach. It was demonstrated that ectopic expression of a membrane associated isoform of M-CSF in GBM cells elicited an anti-tumor response along with TAMs infiltration in a rat intracranial model [102], suggesting that M-CSF may re-activate TAMs into a tumor suppressive phenotype. Administration of lipopolysaccharide (LPS), a chemical that can prime monocytes into mature macrophages *in vitro*, extended survival of mice implanted with intracranial GBMs. Knock-out of TLR-4 in mice antagonized the anti-tumor effect of LPS administration, without changing any histological parameters except for tumor sizes [103]. Since TLR-4 is a key regulator in macrophage activation and no TLR-4 expression is detected in GBM cancer cells, it’s likely that LPS re-activated TAMs via TLR-4 pathway and the activated anti-tumor TAMs in turn suppressed GBM tumor growth. In addition to TLR-4, it had been reported that the TLR-3 agonist poly (I:C) stimulated TAMs to secret toxic cytokines against GBM cell lines *in vitro* [104]. As mentioned above, STAT3 pathway may play a critical role in gain of M2 phenotype by TAMs. Inhibition of STAT3 was found to reverse tolerance in immune cells isolated from GBM patients by induction of the immune-stimulatory cytokines IL-2, IL-4, IL-12, and IL-15 [105,106]. Controversially, oleanolic acid and corosolic acid, two triterpenoid compounds, suppressed the M2 polarization of TAMs according to the inhibition of CD163 expression and IL-10 secretion, along with the activation of STAT3 in both human macrophages and glioblastoma cells [107,108]. Thereby, more studies concerning signaling pathways regulating M2 phenotype TAMs are required to develop more effective strategies for restoration of anti-tumor activities in TAMs.

## 9. Conclusions

Investigation of the infiltrated immune cells in GBMs has become an area of increased study in the past decade. Accumulating evidence suggests that infiltrated immune cells, especially tumor-associated macrophages, are inevitable issues in GBM treatment. This is not only because of the repeatedly observed correlation between the TAM infiltration and the malignant grade of gliomas, but also the concurrence between GBM relapses and TAM re-infiltration. However, so far our knowledge about TAMs in GBMs is very limited. For example, there’s not even a universally accepted marker that can distinguish TAMs from other infiltrated or non-infiltrated immune cells in brain, thereby making the definition of TAMs a vague concept that is largely based on its localization in tumors. However, it has been demonstrated that most, if not all, TAMs have a tumor-supportive role, which could be represented by their lacking of phagocytosis and cytotoxicity, along with their secretion of stimulatory soluble factors. Such immunosuppressive, tumor-supportive features can be concluded as M2 phenotype, although the original definitions of M1/M2 macrophages cannot be strictly applied to GBM TAMs. Furthermore, different lines of evidence demonstrated that it is GBM cancer cells that recruit TAMs from peripheral blood but not resident microglia. Also, GBM cancer cells grant the TAMs with the tumor supportive characteristics, thereby forming a reciprocal supportive interplay between glioblastoma and tumor-associated macrophages. Due to the lack of useful tools to isolate the TAMs from GBM patients and to maintain the TAMs *in vitro* without altering their unique tumor-associated features, few advances have been made concerning the underlying mechanisms regulating TAM differentiation, proliferation, invasion and tumor supportive and/or tumor suppressive functions. However, the development of rodent GBM models along with the molecular manipulation of relevant genes in GBM tumor cells and the mouse genomes will make the in-depth study possible for TAMs in GBMs in the near future. The mechanistic studies as well as the translational research targeting TAMs are of after minor revision utmost importance to explore new therapeutic approaches for GBM treatment.
